# Systematic Review of Ligament Reconstruction of Traumatic Isolated Thumb Carpometacarpal Joint Dislocation

**DOI:** 10.5435/JAAOSGlobal-D-22-00103

**Published:** 2022-12-05

**Authors:** Tannor Court, Kumail Hussain, Jennifer Sohjeong Kim, Ishan Patel, Devan O. Higginbotham, Andrew G. Tsai

**Affiliations:** From the Department of Orthopaedic Surgery, Detroit Medical Center, Detroit, MI (Dr. Court, Dr. Patel, Dr. Higginbotham, and Dr. Tsai), and the Chicago Medical School, Rosalind Franklin University of Medicine and Science, North Chicago, IL (Hussain and Kim).

## Abstract

Thumb carpometacarpal (CMC) joint dislocations are rare with minimal agreement on surgical management. The dorsoradial ligament (DRL) is the primary joint stabilizer but has not historically been reconstructed. We hypothesize that the reported reconstruction of first CMC joint dislocations primarily uses the flexor carpi radialis (FCR) without collective agreement on a surgical technique.

**Methods:**

A systematic review of the PubMed database from 1996 to 2022 was done. Keywords were “thumb dislocation,” “thumb carpometacarpal dislocation,” and “carpometacarpal joint ligament repair.” Inclusion criteria included isolated, unstable thumb CMC dislocations with reconstruction. The Preferred Reporting Items for Systematic Reviews and Meta Analyses guidelines were used.

**Results:**

Four hundred thirty-seven records were identified, and nine met inclusion criteria. Two articles were cohort studies, and seven were case reports. Thirty-seven patients were included, and 26 patients had reconstruction with tendonous autograft. Twenty-five (96.2%) used the FCR and 1 (3.9%) from the palmaris longus. Three patients had reconstruction with a suture anchor. Surgical techniques varied between studies.

**Discussion:**

The recommendation of the authors recreates the DRL during autograft repair. Current repair techniques that recreate the DRL use the FCR, but quantitative comparisons of tendonous autografts or suture anchors have not been done.

The thumb carpometacarpal (CMC) joint is a synovial, biconcave saddle joint, with its stability originating from the native alignment of the joint, surrounding ligaments, and capsule. The unique anatomy confers a highly mobile joint important for hand function, but it is inherently unstable and prone to injury.^1^ The joint stability comes from 16 total ligaments, but only five of these provide most of the stability to the joint. These ligaments include the anterior oblique ligament (AOL), posterior oblique ligament, dorsoradial ligament (DRL), ulnar collateral ligaments, and the first intermetacarpal ligaments, with the DRL being the major contributor (Figure 1).^2,3,4,5,6,7^ Specifically, the DRL has been shown to be the main restraint to dorsal subluxation in traumatic conditions.^8^

**Figure 1:**
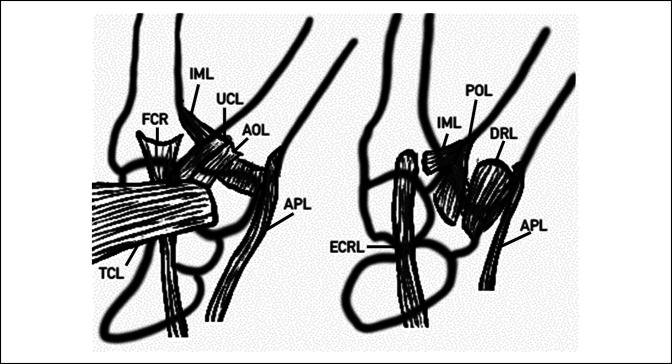
Illustration showing the major stabilizing structures of the first CMC joint. Volar (left) and dorsal (right) illustrations of the main stabilizers and correlated structures of the first CMC joint. AOL = anterior oblique “beak” ligament, APL = abductor pollicis longus, CMC = carpometacarpal, DRL = dorsoradial ligament, ECRL = extensor carpi radialis longus, FCR = flexor carpi radialis longus, IML = intermetacarpal ligament, TCL = transverse carpal ligament, UCL = ulnar collateral ligament

Isolated dislocations of the CMC joint are rare and account for less than 1% of all hand injuries.^9,10^ These injuries are commonly due to acute trauma without identified risk factors, but the presence of previous laxity may act as one inherent risk factor for low-energy traumatic dislocations. Ligamentous laxity of the first CMC joint can be due to previous trauma or subluxation leading to hypermobility.^2^ Due to the high energy of most of these injuries, fracture dislocations are more common than pure ligamentous injuries.^11^ This makes recommendations for the management and literature of isolated first CMC dislocations sparce.

The initial management of first CMC dislocations begins with closed reduction and immobilization; this method can be definitive if the joint is stable postreduction.^12^ If postreduction films show an unstable or inadequately reduced joint, these injuries should be treated with closed reduction and percutaneous pinning with Kirschner wire fixation to maximize joint stability. However, adequate reduction of these injuries depends on the severity of the injury, and due to the intrinsics of the joint, instability after reduction and/or percutaneous pinning is highly likely. Without this, persistent joint instability after reduction can lead to articular degeneration, chronically impairing normal hand function.^13^ Furthermore, an unstable first CMC joint can reduce the function of the thumb, which is used for the most handling maneuvers. Loss of thumb function has been shown to contribute up to a 50% rate of upper extremity impairment.^14^ For these reasons, injury to any of the major ligaments contributing to joint stability should be repaired because neglected dislocations, incomplete reductions, or residual instability can result in chronic arthritis and secondary pain.^15^

Of note, trauma is not the only cause of chronic instability to the first CMC joint. Other causes of instability include idiopathic ligamentous hyperlaxity or other conditions that affect the laxity of ligamentous structures across the entire body.^2^ Although treatment for first CMC joint instability is similar to the treatment of unstable traumatic dislocations, often first CMC joint instability from other causes will have multidirectional instability by the inherent nature of the pathology affecting the joint, thus changing surgical management strategies.

Eaton and Littler^16^ proposed one of the first surgical techniques to reconstruct the ligaments of the first CMC joint. Their technique, which used a portion of the flexor carpi radialis (FCR) as a tendonous autograft, which is weaved through a bone tunnel in the first metacarpal and around the abductor pollicis longus (APL). This acts to reconstruct the stability of the AOL, DRL, and radial collateral ligaments and to correct the injury hypermobility.^17^ These ligaments were chosen because the initial thought was that the AOL acted as the primary stabilizer of the thumb CMC joint. Since the induction of this surgical technique, many modifications and new procedures were developed to treat first CMC joint instability. Importantly, many of these procedures were created before the conceptual shift to the main stabilizer being the DRL and some after this shift. This has caused multiple techniques to use various tendons, different ligaments being reconstructed, and/or different surgical techniques that have varying clinical outcomes. Due to this, controversy exists as to which the ligament reconstruction technique provides the best outcomes postoperatively regarding pain, range-of-motion (ROM), and grip strength. The purpose of this study was to conduct a systematic review on the current published literature on surgical ligamentous reconstruction for isolated, unstable CMC dislocations to assess the reported outcomes and determine a best-suited surgical technique for management.

## Methods

**Figure 2:**
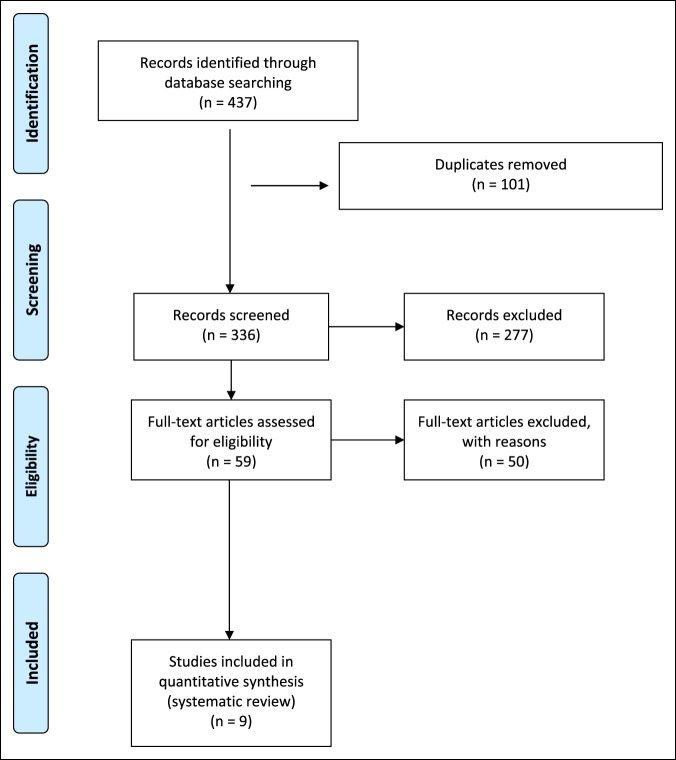
PRISMA diagram. PRISMA = Preferred Reporting Items for Systematic Reviews and Meta-analyses

**Figure 3:**
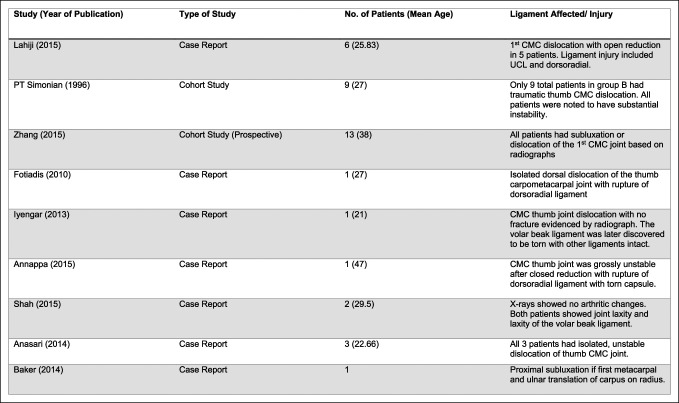
Table showing the description of included studies. CMC = carpometacarpal, UCL = ulnar collateral ligament

**Figure 4:**
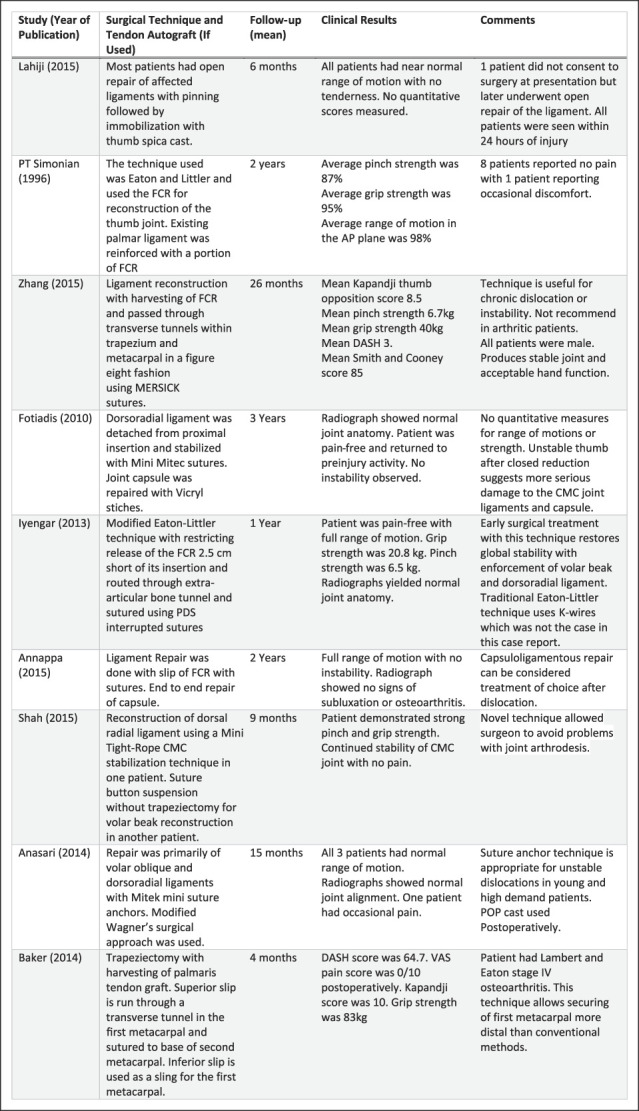
Table showing the outcomes and important notes from included studies. FCR = flexor carpi radialis

MEDLINE Journals were searched using the PubMed database to identify relevant studies pertaining to thumb dislocation from 1996 to 2022. In an attempt to exclude articles that did not recognize the significance of the DRL in thumb CMC stability, keyword searches used were ‘thumb dislocation,’ “thumb carpometacarpal dislocation,” and “carpometacarpal joint ligament repair.” Inclusion criteria included all primary articles in English which described isolated, unstable thumb CMC dislocations and their subsequent surgical restoration in a nonpediatric population. Exclusion criteria included articles without clinical follow-up. Eligibility of the full-length articles for review was assessed based on open surgical repair of damaged ligaments with or without tendon autografts within case reports or cohort studies. Exclusion criteria included articles written in other languages, reviews, nonisolated dislocations, and manuscripts of other joints. The Preferred Reporting Items for Systematic Reviews and Meta Analyses guidelines were used as shown in Figure 2. The data extracted from the studies included number of patients, affected ligaments, tendon used for autograft, previous injury or conditions, procedural technique, and measurable clinical outcomes. The principal outcome measures were stabilization and patient satisfaction, which was defined by ROM, radiograph of joint anatomy, pinch and grip strength, and pain all measured during follow-up. The key studies are described in Figures 3 and 4.

## Results

The search query identified 437 initial records whose abstracts were selected according to the inclusion and exclusion criteria. As shown in Figure 2, a total of 59 full-text articles were selected for review of the full text. Fifty full-text articles did not meet the inclusion criteria and were further excluded, thus leaving a total of nine studies to be used for the literature analysis. Three articles were cohort studies, and seven were case reports. A total of 37 patients were included among the key studies. Most patients described in these articles were younger patients with an average age of 30.94 years, 86.1% were male, and additional demographic information was not included within the included articles. Those with stable lesions were treated conservatively as a first-line approach in most of the cases. For unstable lesions, all studies conducted open reduction for the first CMC joint; however, the surgical techniques varied. In total, 7 of the 37 patients (18.9%) underwent closed reduction without additional surgical management, 3 of the 37 (8.1%) used a suture anchor technique, the remaining 26 of the 37 (70.3%) underwent open reduction with the use of a tendon autograft. Of these patients, 25 (96.2%) used the FCR and 1 (3.9%) the palmaris longus (PL). Slips of tendon autograft were typically run transversely through the first metacarpal to allow for stabilization of the CMC joint, with or without utilization of nearby tendon stabilization. Reported ligaments affected by the injuries were the DRL, AOL, and volar beak ligaments. All but one study indicated the dorsoradial, and volar beak ligaments were the reconstructed ligaments. Variable uses of sutures were reported for the repair of the joint capsule: One case with PDS interrupted sutures, one case with 4.0 MERSICK sutures, or repair was not specified. Postoperatively, immobilization was done with a thumb spica cast, a thumb spica splint, and one article used a plaster of Paris cast. The success of the treatment was assessed by different criteria: Disabilities of the Arm, Shoulder, and Hand (DASH) and Kapandji thumb opposition scores, pinch and grip strength, or radiographs to assess for instability. Nearly all articles inquired about any pain and ROM at follow-up from the procedure. Most procedures were well tolerated, complications included one case with persistent pain that did not interfere with daily hand function.

## Discussion

The selected literature describes cases in which patients had undergone ligamentous reconstruction due to residual instability after closed reduction, acute subluxation, or chronic instability. Due to the rarity of this condition, few articles specified which patients fell into each of these categories. However, the current literature provided does seem to indicate first CMC dislocations, as a whole, occur more commonly within the young male population. However, no epidemiologic studies have been done to confirm this.

Among the included articles, controversy still exists as to which ligaments stabilize the CMC joint, likely leading to the variation in which ligaments are being reconstructed/repaired. Older articles have frequently reported the AOL as the primary stabilizer of the first CMC joint, but newer studies have revealed it is most likely the DRL acting as the main stabilizer of the thumb.^18,19^ The original Eaton Littler technique for repair assumed the AOL as the main stabilizer for first CMC dislocations, with the new finding of the DRL acting as the main stabilizer contradicting previous research and is likely the reason why the surgical technique has begun to vary so drastically.^18^ A commonly cited case report from Shah et al^20^ directly demonstrated two separate surgical procedures in two different patients in which the AOL or the dorsal ulnar ligament was reconstructed. The results favored reconstruction and repair of the dorsal ulnar ligament which provided a satisfactory outcome for the patient with continued CMC joint stability and no pain, but no direct discussion was made about the DRL.^20^

Due to the shift in focus from reconstructing the AOL to DRL or other ligaments, modified Eaton-Littler techniques are being used through the FCR, APL, PL, suture anchors or self-locking extracapsular suture device. In the setting of trauma, only the use of the FCR and PL have been described about ligamentous autografts. Regarding the use of the FCR, one technique used a modified Eaton-Littler reconstruction. In this technique, the part of the FCR was directed as a slip in a more oblique manner to reproduce volar and DRL stability. This technique proved to be successful in providing global stability of the thumb and reinforcing the AOL and DRL, with a clinically significant increase in pinch and grip strength.^21^ A similar technique used a two-bone tunnel technique, and the FCR was passed through the trapezium and the metacarpal from the radial side to the ulnar side. This technique recreated the AOL with an increased radial and ulnar stability. Postoperatively, the patients did well with no residual instability and clinically sufficient grip strength.^17^ In a simpler technique, the FCR was passed under the APL and fixed into place with a Kirschner wire and sutures, reinforcing the DRL. This technique provided adequate pain control for long-term and full ROM, and radiograph findings showed successful reduction after 2 years of follow-up.^22^ These articles demonstrated the favorable outcomes with the harvesting of the FCR tendon. Notably, the original Eaton-Littler technique in which the slip of FCR is passed through the first metacarpal provides satisfactory stability of the joint with improvement of grip strength, pinch strength, and ROM postoperatively.^23^ However, the dorsal aspect of the CMC joint has to be extensively exposed and increases the risk of iatrogenic injury.^2^ Many of these surgical techniques are variants of the original Eaton-Littler technique and have been improved to minimize incision size, size of ligamentous excision, and loss of radial nerve sensation and to reproduce actions of the torn ligaments.^21^

Another study showed the use of the PL tendon as an autograft in a first CMC dislocation. Importantly, this patient was shown to have notable osteoarthritis which resulted in their chronic subluxation. Due to this, a trapeziectomy was initially done followed by the use of a transverse hole through the first metacarpal. This hole is more distal than the previously described techniques due to it being a free tendon. This tendon was then positioned as a sling through the bone tunnel attaching to the base of the second metacarpal. This reconstruction focused on recreating the AOL. The patient was followed up with 4 months after and demonstrated a DASH score of 6.2, mean VAS score at rest of 1.1, and mean Nelson score of 87.7, showing a notable improvement in grip and pinch strength, and a 0/10 VA pain score.^24^ Other studies have used different autografts such as the APL to treat chronic first CMC instability, but not in the direct setting of unstable traumatic dislocations.^2^

Finally, new techniques have begun to focus on the recreation of the CMC joint using nonautograft repairs. Specifically, one technique used a suture anchor that directly recreated the function of AOL and the DRL. This technique showed good clinical results after 15 months. Clinically, functional capacity was restored in all treated patients, but one of the three reported mild intermittent pain that did not affect hand function or quality of life.^25^

All these mentioned studies show a successful reconstruction of the ligamentous stability for the CMC joint. However, despite current literature focusing on the importance of the DRL as the primary stabilizer in first CMC dislocations, many of these described techniques do not recreate its anatomic function directly. Only three of the mentioned studies directly attempted to recreate this ligament. These included the Modified Eaton-Littler's Reconstruction, that showed that recreation of the volar and DRL stability can successfully treat first CMC dislocations, the Kirschner wire fixation of the FCR to reinforce the disrupted DRL that showed excellent postoperative results, and the suture anchor technique. It should be noted that no direct scoring system was assessed in the Kirschner wire fixation or the suture anchor technique.

Few of the mentioned studies used a standardized method of assessing patient postoperative function, but all the studies showed notable clinical improvement with minimal complications. Overall, based on current biomechanical data, it is the authors’ recommendation to focus tendonous reconstruction on recreating the function of the DRL. Additional ligamentous stability can be used in techniques such as the Modified Eaton-Littler. Ligamentous autograft using the FCR is the most commonly reported tendon used in first CMC dislocations, but no quantitative comparisons or direct clinical reporting exists to support the use of one tendon over another. The currently described techniques use the FCR to recreate the DRL function, but future studies must be done to isolate a clinically superior autograft and to directly compare the use of autograft to suture anchors or self-locking extracapsular suture device repairs.

## Conclusion

The goal of this literature review was to analyze the open repair techniques and compare the treatment measures for unstable dislocations of the first CMC joint. The mechanism of injury in patients varied widely but mostly included CMC dislocation or subluxation and damage to the anterior oblique ligament or DRL. No universally accepted method of assessing the stability of the thumb joint after dislocation exists. However, scores such as DASH, VAS, or Kapandji thumb opposition scores currently serve as the most objective measurements of thumb function. Therefore, these types of scores should be used more often to allow for more accurate comparisons in long-term treatment. A notable percentage of patients underwent open reduction with the use of a tendon autograft or the use of a suture anchor to recreate ligamentous stability. If an autograft was used, the open recreation of damaged ligaments most commonly used the FCR, given its proximity to the lesion and its inherent durability, one reported case used the PL. Due to the DRL functioning as the major ligamentous stabilizer preventing dislocation, it is the author's recommendation to focus on recreating its function with open repair of these injuries. Due to a lack of clinical or quantitative data directly comparing tendonous autografts used in the repair of these injuries, no direct recommendation can be made on which tendon to use. However, it should be noted that the current techniques that recreate the function of the DRL use the FCR as the autograft of choice.

## References

[1] McCarley M Foreman M: Chronic carpometacarpal dislocation of the thumb: A case report and review of the literature. JBJS Case Connect 2018;8:e49. 29995663 10.2106/JBJS.CC.17.00206

[2] Stauffer A Schwarz Y Uranyi M : Outcomes after thumb carpometacarpal joint stabilization with an abductor pollicis longus tendon strip for the treatment of chronic instability. Arch Orthop Trauma Surg 2020;140:275-282. 31691837 10.1007/s00402-019-03302-8 PMC6989670

[3] Imaeda T An KN Cooney WP III Linscheid R: Anatomy of trapeziometacarpal ligaments. J Hand Surg 1993;18:226-231. 10.1016/0363-5023(93)90352-4 8463585

[4] Strauch RJ Behrman MJ Rosenwasser MP: Acute dislocation of the carpometacarpal joint of the thumb: An anatomic and cadaver study. J Hand Surg 1994;19:93-98. 10.1016/0363-5023(94)90229-1 8169374

[5] Bettinger PC Linscheid RL Berger RA Cooney WP III An KN: An anatomic study of the stabilizing ligaments of the trapezium and trapeziometacarpal joint. J Hand Surg 1999;24:786-798. 10.1053/jhsu.1999.0786 10447171

[6] Colman M Mass DP Draganich LF: Effects of the deep anterior oblique and dorsoradial ligaments on trapeziometacarpal joint stability. J Hand Surg 2007;32:310-317. 10.1016/j.jhsa.2006.12.002 17336836

[7] Van Brenk B Richards RR Mackay MB Boynton EL: A biomechanical assessment of ligaments preventing dorsoradial subluxation of the trapeziometacarpal joint. J Hand Surg 1998;23:607-611. 10.1016/s0363-5023(98)80045-2 9708373

[8] Bosmans B Verhofstad MHJ Gosens T: Traumatic thumb carpometacarpal joint dislocations. J Hand Surg 2008;33:438-441. 10.1016/j.jhsa.2007.11.022 18343304

[9] Kim JS Hussain K Higginbotham DO Tsai AG: Management of thumb carpometacarpal joint dislocations: A systematic review. J Orthop 2021;25:59-63. 33927510 10.1016/j.jor.2021.03.015 PMC8065249

[10] Mueller JJ: Carpometacarpal dislocations: Report of five cases and review of the literature. J Hand Surg 1986;11:184-188. 10.1016/s0363-5023(86)80048-x 3958446

[11] Alexander C Abzug JM Johnson AJ Pensy RA Eglseder WA Paryavi E: Motorcyclist's thumb: Carpometacarpal injuries of the thumb sustained in motorcycle crashes. J Hand Surg Eur Vol 2016;41:707-709. 26642850 10.1177/1753193415620186

[12] Slocum AMY Lui TH: Isolated first carpometacarpal joint dislocation managed with closed reduction and splinting. BMJ Case Rep 2019;12:e228715. 10.1136/bcr-2018-228715 PMC6453362 30936354

[13] Jeong C Kim HM Lee SU Park IJ: Bilateral carpometacarpal joint dislocations of the thumb. Clin Orthop Surg 2012;4:246-248. 22949958 10.4055/cios.2012.4.3.246 PMC3425657

[14] Pellegrini VD Jr: The ABJS 2005 Nicolas Andry Award: Osteoarthritis and injury at the base of the human thumb: Survival of the fittest? Clin Orthop Relat Res 2005;438:266-276. 16131901 10.1097/01.blo.0000176968.28247.5c

[15] Lahiji F Zandi R Maleki A: First carpometacarpal joint dislocation and review of literature. Arch Bone Joint Surg 2015;3:300-303. 26550598 PMC4628640

[16] Eaton RG Littler JW: Ligament reconstruction for the painful thumb carpometacarpal joint. J Bone Joint Surg Am 1973;55:1655-1666. 4804988

[17] Zhang X Shao X Huang W Zhu H Yu Y: An alternative technique for stabilisation of the carpometacarpal joint of the thumb after dislocation or subluxation. Bone Joint J 2015;97-B:1533-1538. 26530657 10.1302/0301-620X.97B11.35482

[18] Fotiadis E Svarnas T Lyrtzis C Papadopoulos A Akritopoulos P Chalidis B: Isolated thumb carpometacarpal joint dislocation: A case report and review of the literature. J Orthop Surg Res 2010;5:16. 20219137 10.1186/1749-799X-5-16 PMC2841114

[19] Owings FP Calandruccio JH Mauck BM: Thumb ligament injuries in the athlete. Orthop Clin North Am 2016;47:799-807. 27637666 10.1016/j.ocl.2016.06.001

[20] Shah A Martin G III Thomson JG: A novel use for suture button suspension: Reconstruction of the dorsal ulnar ligament to treat thumb metacarpal dislocation. Case Rep Plast Surg Hand Surg 2015;2:7-11. 10.3109/23320885.2014.997823 PMC4623541 27252958

[21] Iyengar K Gandham S Nadkarni J Loh W: Modified Eaton-Littler's reconstruction for traumatic dislocation of the carpometacarpal joint of the thumb-A case report and review of literature. J Hand Microsurg 2013;5:36-42. 24426670 10.1007/s12593-012-0067-x PMC3650165

[22] Annappa R Kotian P Janardhana Aithala P Mudiganty S: Ligamentous reconstruction of traumatic dislocation of thumb carpometacarpal joint: Case report and review of literature. J Orthop Case Rep 2015;5:79-81. 27299108 10.13107/jocr.2250-0685.354 PMC4845467

[23] Simonian PT Trumble TE: Traumatic dislocation of the thumb carpometacarpal joint early ligamentous reconstruction versus closed reduction and pinning. J Hand Surg 1996;21:802-806. 10.1016/S0363-5023(96)80195-X 8891977

[24] Baker RHJ Sood MK: A new technique of first carpometacarpal joint suspension arthroplasty with palmaris longus tendon graft. Tech Hand Up Extrem Surg 2014;18:98-101. 24667687 10.1097/BTH.0000000000000045

[25] Ansari MT Kotwal PP Morey VM: Primary repair of capsuloligamentous structures of trapeziometacarpal joint: A preliminary study. J Clin Orthop Trauma 2014;5:185-192. 25983496 10.1016/j.jcot.2014.09.009 PMC4264062

